# A149 LYTIC PHAGE REDUCES COLITIS SEVERITY IN MICE COLONIZED WITH IBD-ASSOCIATED *E. COLI* NRG857C

**DOI:** 10.1093/jcag/gwae059.149

**Published:** 2025-02-10

**Authors:** K Jackson, H Galipeau, A Hann, M Zangara, M Bording-Jorgensen, B Coombes, Z Hosseinidoust, E Verdu

**Affiliations:** Chemical Engineering, McMaster University Faculty of Engineering, Hamilton, ON, Canada; McMaster University Faculty of Health Sciences, Hamilton, ON, Canada; McMaster University Faculty of Health Sciences, Hamilton, ON, Canada; McMaster University, Hamilton, ON, Canada; McMaster University Faculty of Health Sciences, Hamilton, ON, Canada; McMaster University, Hamilton, ON, Canada; Chemical Engineering, McMaster University Faculty of Engineering, Hamilton, ON, Canada; McMaster University Faculty of Health Sciences, Hamilton, ON, Canada

## Abstract

**Background:**

Current therapies for inflammatory bowel disease (IBD) suppress inflammation rather than inhibiting the underlying drivers. Treatment failures can lead to dose escalation and risk of adverse effects. Bacteria with pathogenic potential, like adherent-invasive *Escherichia coli* (AIEC), have been postulated as one possible microbial driver in Crohn’s disease (CD), however, targeting these bacteria with precision in IBD remains challenging. Most antibiotics have broad-spectrum activity and disturb the background microbiome, which may exacerbate inflammation.

**Aims:**

We aimed to assess whether and how bacteriophage, specific for a CD-associated bacterium, improves colitis severity in gnotobiotic mouse models.

**Methods:**

Adult germ-free C57BL/6 mice were colonized with altered Schaedler-flora (ASF) and *E. coli* NRG857c (NRG), a CD-associated isolate. After 3 weeks, mice were treated with an NRG-specific lytic phage (1x10^9^ PFU/dose; daily or TIW) or vehicle (PBS) for 2 weeks (n=6/group) and then exposed to 1 cycle of dextran sulfate sodium (2%; DSS) in drinking water. Additional PBS-treated mice (n=6) not exposed to DSS served as controls. Phage treatment was also tested in a spontaneous model of colitis using C57BL/6NTac-*Il10*^*em8Tac*^ (IL-10^-/-^) mice, treated weekly with phage. In both models, mice were monitored daily for weight, stool consistency, and occult blood. Fecal contents were cultured to determine bacterial load, and the expression of NRG *fimS* was quantified using qPCR as an assessment of bacterial virulence. At endpoint, colon tissues were collected for histological and immunohistochemistry analysis to quantify colitis severity and NRG infiltration, respectively.

**Results:**

In ASF and NRG colonized mice, phage treatment reduced clinical symptoms (p< 0.001, p<0.001) and histological scores (p< 0.0001, p< 0.001) of chemical and spontaneous colitis respectively. A1-log reduction in NRG bacterial load was observed in phage-treated mice (p< 0.001), and reisolated NRG exhibited a reduced FimS ON/OFF ratio (p-value < 0.01). Immunohistochemistry revealed reduced infiltration of NRG into the lamina propria following phage treatment, with greater accumulation in the epithelium.

**Conclusions:**

Our results demonstrate that lytic phage targeting a CD-associated bacterium attenuates colitis in two gnotobiotic mouse models. Whereas this model did not lead to complete eradication of the disease-driving agent, phage treatment was associated with a reduction in a known AIEC virulence factor, attenuating colitis severity. The data suggest alternative mechanisms should be considered when translating phage therapy to treat IBD in clinical trials.

**Funding Agencies:**

CIHRFarncombe Innovation Fund

